# Genetic investigations into the use of sensory evaluation: the case of boar taint discrimination in Pietrain sired crossbreds

**DOI:** 10.1093/jas/skae389

**Published:** 2024-12-24

**Authors:** Alice Markey, Christine Groβe-Brinkhaus, Daniel Mörlein, Johanna Mörlein, Hélène Wilmot, Ernst Tholen, Nicolas Gengler

**Affiliations:** TERRA Teaching and Research Center, University of Liège, Gembloux Agro-Bio Tech (ULiège-GxABT), 5030, Gembloux, Belgium; Institute of Animal Science, University of Bonn, 53115, Bonn, Germany; Department of Animal Sciences, University of Göttingen, 37077, Göttingen, Germany; Department of Animal Sciences, University of Göttingen, 37077, Göttingen, Germany; Department of Animal Sciences, University of Göttingen, 37077, Göttingen, Germany; TERRA Teaching and Research Center, University of Liège, Gembloux Agro-Bio Tech (ULiège-GxABT), 5030, Gembloux, Belgium; National Fund for Scientific Research (F.R.S.-FNRS), 1000, Brussels, Belgium; Institute of Animal Science, University of Bonn, 53115, Bonn, Germany; TERRA Teaching and Research Center, University of Liège, Gembloux Agro-Bio Tech (ULiège-GxABT), 5030, Gembloux, Belgium

**Keywords:** androstenone, human nose score, meat quality, phenotyping, recursive model, skatole

## Abstract

Using genetic selection for raising intact boars, which improves growth and feed efficiency, is a promising alternative to castration for mitigating boar taint. Selective breeding has the potential to help to identify and select genetic lines with a reduced risk of boar taint. Common phenotypes are laboratory measurements of skatole (**SKA**) and androstenone (**ANON**) i.e., the major compounds responsible for boar taint, in backfat. However, an alternative exists: sensory evaluation by human assessors. The objectives of this study were (1) to estimate the genetic relationships among sensory scores (**SENS**) obtained by different assessors, (2) to correlate these scores with SKA and ANON, (3) to establish the independence of SENS from the causal traits, here SKA and ANON, by recursive modeling, holding those constant, and (4) to combine different assessors to allow an efficient selection against boar taint. Data included up to 1,016 records of SKA, ANON, and SENS (0–5) from 10 trained assessors on the backfat of intact males reared at least until puberty at three performance testing stations testing the products of Pietrain × commercial crossbred sows. Genetic parameters were estimated using restricted estimate maximum likelihood. Traits SKA and ANON were log_10_ transformed (SKAt and ANONt) and SENS traits were Snell transformed SENS (SENSt). Heritability estimates were 0.52 for SKAt and 0.53 for ANONt, those for SENSt ranged from 0.07 to 0.30. Moderate to high genetic correlations between some SENSt and SKAt (up to 0.87) and ANONt (up to 0.61) were found. Heritabilities and correlations indicated that some SENSt could be used to select against boar taint. Studying the independence of SENSt from SKAt and ANONt based on a posteriori recursive model revealed a large range of reductions of genetic variance: up to 71.08%. However, some SENSt remained moderately heritable (0.04–0.19) indicating independent genetic variance from SKAt and ANONt. This reflects that some heritable compounds potentially not related to SKA or ANON are perceived. Finally, the combination of assessors allowed, here shown with three assessors, to obtain a high heritability of 0.40, associated to high genetic and phenotypic correlations. Moreover, these results demonstrate the potential of using the sensory scores of several trained assessors for selection against boar taint.

## Teaser

It seems possible to use sensory evaluations, or combinations of those, as reliable phenotypes for breeding against boar taint risk. In order to draw novel conclusions, we used a recursive model to assess the (in)dependence of sensory scores with respect to the major compounds of boar taint and we identified an optimal combination of assessors for achieving the highest genetic progress.

## Introduction

The castration of piglets is well known to be a painful practice but remains common in many production systems. Raising intact male pigs can be a potential alternative. Uncastrated male pigs have the potential for better growth rates and improved feed efficiency, especially feed conversion into muscles, compared to barrows (Lundström et al., 2009; Duarte et al., 2021). Despite these advantages, there are several drawbacks to non-castration, e.g., more aggressive animals and, above all, the possible development of boar taint (Parois et al., 2015; Bonneau and Weiler, 2019). Therefore, a large part of the industry still performs castration. For animal welfare reasons, the European pig industry is looking for alternatives to surgical castration of piglets that, even when performed under anesthesia, remains a time-consuming, costly, and post-operatory painful process for piglets. Castration can also lead to infections and impaired growth (Bonneau and Weiler, 2019). Moreover, some countries have already forbidden castration without pain relief, including Germany since 2021 (Deutscher Bundestag, 2018; Brinke et al., 2022) and France since 2022 (Ministère de l’agriculture et de l’alimentation, 2021). Considering the abolition of castration, the risk of boar taint can generate economic losses for producers due to unpleasant consumer experiences, since this taint is associated with fecal and urinary odors. In this context, the detection and mitigation of boar taint in slaughtered intact males becomes an important issue as it is the cornerstone for supporting the use of uncastrated males.

Boar taint primarily arises due to elevated levels of skatole (**SKA**) and androstenone (**ANON**) which accumulate mainly in the fat. This unpleasant and commercially highly undesirable odor is emitted upon cooking. Consumers may perceive the odor to various degrees, depending on their olfactory acuity. Moreover, some people do not perceive ANON as an unpleasant taint (i.e., estimated around 50% of the European population), on the opposite, SKA is widely perceived as unpleasant when presented in relevant amounts (Weiler et al., 2000; Bekaert et al., 2011; Borrisser-Pairó et al., 2017; Heyrman et al., 2020). ANON is synthesized in Leydig cells and its production is part of the steroid cycle whereas SKA is generated as a result of bacterial activity in the gut of pigs (Wesoly and Weiler, 2012; Robic et al., 2014; Squires et al., 2020). Moreover, SKA levels are also influenced by ANON regulation (Zamaratskaia and Squires, 2009).

As mentioned, even if the use of entire male pigs may be desirable in terms of welfare and feed conversion, it is of main importance to minimize the risk of boar taint by reducing the risk of appearance of these compounds in the meat. Breeding efforts aiming at mitigating these compounds have demonstrated considerable potential as SKA and ANON are known to have moderate to high heritabilities (ranging from 0.23 to 0.55 for SKA and ranging from 0.55 to 0.88 for ANON) as reported by Duarte et al. (2021). A simulation was performed by Haberland et al. (2014) estimating that boar taint could be minimized by up to 50% after 6–8 yr of selection. Yet, focusing selection only on SKA and ANON still has several major issues. First, these compounds must be quantified: many methods exist, and new ones are still under development (Font-i-Furnols et al., 2020; Burgeon et al., 2021a). However, analytical laboratory methods are costly and time consuming, and do not allow the current daily assessment of tainted animals at the slaughter line. Research is focusing on these disadvantages by developing sensors and fast gas chromatography methods for example (Verplanken et al., 2017; Burgeon et al., 2021a). Furthermore, thresholds must be used to define tainted and untainted samples based on SKA and ANON levels. Finally, even though SKA and ANON are considered to be the main taint compounds, this does not exclude the existence of others (Burgeon et al., 2021a).

Nevertheless, the main challenge lies in identifying boar taint levels perceptible by humans, leading to the use of sensory evaluation, also called human nose method in the industry. In practice, a trained line operator, i.e., a slaughterhouse technical employee, heats the backfat and an assessor indicates the presence or absence of boar taint. This method can also be used on fat samples, at-line, in a laboratory at the slaughterhouse, or off-line, in an independent laboratory (Font-i-Furnols et al., 2020). The ability of this method to generate relatively large datasets renders it appealing for breeding purposes. In previous studies, the heritability estimates of the sensory evaluation, that is, sensory scores obtained by trained sensory assessors (**SENS**), were reported to be between 0.11 and 0.19 (Windig et al., 2012; Mathur et al., 2013). However, studies have shown that chemical measurements and SENS do not always agree (Meier-Dinkel et al., 2015; Mörlein et al., 2016) even though assessors were carefully selected and trained to detect boar taint compounds SKA and ANON.

The main objective of this study was to investigate the use of sensory phenotyping for breeding against boar taint. First, the trained assessors were evaluated for their sensory performances, that is, validity tests to assess their accuracy and other performances. Second, the variance components of their scores were estimated. Thirdly, the independence towards SKA and ANON of SENS was studied using a recursive modeling approach. This allowed us to characterize the direction of the relation between traits without the influence of SKA and ANON. Finally, in the context of the genetic evaluation of sires through progeny testing, the optimized combination of several sensory assessors was evaluated.

## Materials and Methods

### Ethics statement

All applicable international, national, and institutional guidelines for the care and use of animals were followed. Animals were reared in performance testing stations of two German herdbook organizations (SZV and EGZH) in compliance with national regulations pertaining to livestock production and according to the procedures approved by the German Animal Protection Law (TierSchG). These animals are not to be considered experimental animals as defined in European Union directive 2010/63 and the Animal Protection Law (TierSchG). The investigated phenotypic data used for the current study has been already published in Mörlein et al. (2016). As the data were available from routine breeding management, there was no need for ethical approval of this.

### Animals

This study was based on experimental data from Mörlein et al. (2016). Our study included 1,016 animals with available records with observed traits being SKA, ANON, and SENS ratings, and known pedigree. Major elements of sample collection and measurements are given hereafter; more details can be found in the phenotypic study of Mörlein et al. (2016). All required authorizations were obtained, and sample collection was included in a normal performance testing process.

### Sample collection

Entire males produced through the mating of Pietrain paternal lines with crossbred commercial sows (Pi × F1) were reared at three performance testing stations in Germany. The same protocol was used on all three stations and animals were slaughtered at approximately the same carcass weight of around 90 kg and on average at 176 d (ZDS, 2007). Backfat samples were collected at the slaughterhouse, then divided into two subsamples, and were stored at −18 °C until analyses.

### Phenotyping boar taint traits

Chemical analyses were based on the method developed by Fischer et al. (2011) that quantified simultaneously SKA (3-methylindole) and ANON (5α-androst-16-en-3-one) by stable isotope dilution-headspace solid-phase microextraction-gas chromatography-mass spectrometry (**SIDA-HS-SPME-GC-MS**) with internal deuterium-labeled standards. The method was validated by variation coefficients of the analyzed compounds of <10%.

Sensory evaluation was performed using a human sensory panel scoring system. SENS were extracted from sensory panel measurements in Mörlein et al. (2016) and consisted of SENS ratings obtained by 10 trained assessors on 1,016 backfat samples. These assessors were selected in a few steps. First, their ability to perceive SKA and ANON was validated, considering possible anosmia for ANON. Then, they were trained to score fats according to SKA and ANON perception. In addition, they were trained to recognize the levels of SKA and ANON. The sensory evaluation consisted of the classification of samples heated in a microwave oven (450 W) for 80 s. Evaluations by assessors were based on a 6-point scale. They characterized the perceived deviation from a standard sample from 0 (= nondeviant odor) to 5 (highly deviant odor). More details about the sensory method are available in Mörlein et al. (2016). In the original dataset, certain SENS measurements were available for a limited number of observations for two assessors which were excluded. Repeated measurements on the same animal were excluded too. In consequence, eight assessors performed the measurements on all 1,016 samples, two evaluated only 856 samples (Mörlein et al., 2016).

### Data transformation and statistics

Statistics and data transformation were performed using version R4.4.2. of R on RStudio (R Core Team, 2022). As for this study linear models were used, analyzed traits (i.e., ANON and SKA), needed to have an approximately normal distribution and to be continuous. The distribution of ANON and SKA is known to be skewed (e.g., Tajet et al., 2006), therefore a log_10_ transformation was used to reduce this problem and resulted in ANONt and SKAt. The descriptive statistics are available in Supplementary Table S1.

In addition to not being normally distributed and not being continuous, distributions of the SENS were expected to be different from one assessor to another (Mörlein et al., 2016). In order to normalize the distributions, and considering the uneven frequency distributions of scores assigned by each assessor, these scores were transformed to Snell scores using a procedure based on Snell (1964) and implemented by Mujibi and Crews (2009). This normalization can be considered as a simple approximation of the generation of underlying normal data through threshold models. The principle was to replace the SENS, that are, the order numbers of six categories (0–5), by scores representing the 6 classes of observations. For the four bounded classes (scores 1–4), the Snell score representing each class was estimated from the cumulative frequencies of the class boundaries transformed into *z*-scores and then averaged. For the extreme classes (scores 0 and 5), the SENS were replaced by approximations that removed or added -ln(Pe)/Qe to the first or last class, where Pe is the probability of a value below the first or last boundary and Qe is the relative proportion of SENS in these extreme classes (Mujibi and Crews, 2009). Means and SD of transformed SENS (SENSt) were computed. The approach implemented in this study can be considered similar to the one used by Mathur et al. (2012).

Panel performances were evaluated considering SENS (i.e., boar taint perception) as test results and chemical analyses as the gold standard as proposed by Mörlein et al. (2016). According to them, chemical measurements were converted into binary values (0 and 1) whenever at least one of the SKA (0.2 ppm) or ANON (1.5 ppm) rejection thresholds was reached. In other words, in the binary scale, the sample was classified as 0 if both concentrations of SKA and ANON were simultaneously below the rejection thresholds indicated above respectively. If one of the concentrations was above the rejection threshold for the respective compound, then the binary score 1 was assigned. Binary classifications as tainted were applied to SENS ≥ 2. (Dis-)agreement between methods led to classify measurements as true-positives (**TP**), true-negatives (**TN**), false-positives (**FP**), or false-negative (**FN**). The five computed performances of each assessor are sensitivity (TP/[TP + FN]) and specificity (TN/[FP + TN]) which represent respectively the tainted and untainted animals rates declared by assessors; precision (i.e., positive predictive value, TN/[TP + FP]) and negative predicted value (TN/[FN + TN]) which are the probabilities to correctly declare tainted and untainted animals, respectively; and accuracy ([TP + TN]/[TP + TN + FP + FN]), that is, agreement between boar taint perception and gold standard.

### Genetic analyses

The genetic analyses done in this study were twofold. First (co)variance components were estimated using the BLUPF90 family of programs (Misztal et al., 2014). Variance components and derived parameters (heritability, residual, and genetic correlations) were computed using a multivariate linear mixed model associated to restricted maximum likelihood (**REML**) implemented using expectation maximization-REML (**EM-REML**) (Dempster et al., 1977) and average information-REML (**AI-REML**) algorithms (Gilmour et al., 1995). The SE was derived following Meyer and Houle (2013).

Based on the notation presented in the studies of Gianola and Sorensen (2004) and Varona and González-Recio (2023), the recursive model, for the jth multivariate record presented below, is as follows:


$$\begin{array}{l} y_{j}=X_{j}b+u_{j}+e_{j} \end{array}$$

where **b** is the vector of fixed effects (testing station × month of slaughter) and of coefficients of regressions applied to the slaughter weight and the age at slaughtering (94.9 ± 4.81 kg and 175.9 ± 11.31 d—data available for all animals), $y_{j}$, $u_{j}$, and $e_{j}$ are m × 1 vectors of phenotypic measurements, additive genetic effects and residuals of the m traits associated with the jth multivariate record, and $X_{j}$ is its corresponding incidence matrix, with m being 10 respectively 12 traits (SKAt, ANONt, and 8, respectively, 10 SENSt). The general model can therefore be written as follows:


$$\begin{array}{l} y=Xb+Zu+e \end{array}$$

where **y**, **u,** and **e** are vectors representing all phenotypic measurements, additive genetic effects, and residuals, and matrices **X** and **Z** are the corresponding full incidence matrices. The pedigree included 1,934 animals in three generations, but only Pietrain sires had a pedigree. As the correlation between age and weight was estimated to be low (0.02), we did not expect collinearity between regressions on weight and age and kept both in the model. A table (Supplementary Table S2) contains fixed solutions for regression coefficients on weight and age and approximate *t* statistics to describe the effect of those. The additive genetic effects and the residual effects are considered distributed according to the multivariate Gaussian distributions: **u** ~ N (**0**, **A** ⊗ **G**) and **e** ~ N (**0**, I ⊗ **R**), where, **A** is the additive relationship matrix of dimension equal to the number of animals in the pedigree, **I** is an identity matrix of dimension equal to the number of records, here assuming all traits being recorded for each animal. Moreover, **G** and **R** are matrices of elementary genetic and residual (co)variances between analyzed traits. A convergence of (co)variance components below 10^−13^ was achieved beyond 1,000 rounds using EM-REML. Large sample standard errors for (co)variances reported as Supplementary Table S3 were obtained by establishing the AI-REML equations at convergence of EM-REML.

Additional matrix computations were done with the program GNU Octave (Eaton et al., 2020). To evaluate the independence of sensory scores attributions to the samples with respect to the perception of SKA and ANON by the assessors, a recursive model, that is, a statistically equivalent model to the multi-trait model, was applied retrospectively by transforming the (co)variance components **G** and **R** (Varona and González-Recio, 2023). This type of model can estimate if SENS were attributed according to SKA or ANON by decorrelating residuals between SENSt and the causal traits, that are, SKAt and ANONt. In the context of a recursive model, the transformed genetic (co)variance matrix expresses the additive genetic relationships between SENSt traits while holding the causal traits constant. Varona and González-Recio (2023) described the transformation of the genetic (**G**) and residual (**R**) (co)variance matrices associated to the multi-trait mixed model previously used to obtain the a posteriori covariance components (i.e., genetic [**G**_*RM*_] and residual [**R**_*RM*_] covariance matrices) associated with the recursive model $R_{RM}=\Lambda \;R\;\Lambda^{\prime }$ and $G_{RM}=\Lambda \;G\;\Lambda^{\prime }$ where **Λ** is a 12 × 12 matrix of recursive model parameters that would transform original traits to recursive transformed trait, with unit coefficients on the diagonal and nonzero coefficients below the diagonal. In our case, the **Λ** matrix describes the residual dependency of each SENSt trait (traits 3–12) on the first (SKAt) and second (ANONt) traits:


$$\begin{array}{l} \Lambda =\left[\begin{array}{lllllllllllllllllllll} \;1 & \;0 & \;0 & \;\ldots & \;0\;0 & \;1 & \;0 & \;\ldots & \;0\;-\lambda_{1\to 3} & \;-\lambda_{2\to 3} & \;1 & \;\ldots & \;0\;\ldots & \;\ldots & \;\ldots & \;\ldots & \;\ldots \;-\lambda_{1\to 12} & \;-\lambda_{2\to 12} & \;0 & \;\ldots & \;1\; \end{array}\right] \end{array}$$

As explained by Varona and González-Recio (2023), the coefficient of **Λ** can be obtained by an “LDL decomposition” (i.e., Cholesky decomposition variant) of $R=\Lambda^{-1}R_{RM}\Lambda^{-1^{\prime }}$. Moreover, to estimate the variation in variance components between models, relative changes of genetic variances of recursive to multivariate model compared to multivariate model were computed.

### Combining assessors for genetic selection

The last objective was to identify the best combination of assessors to improve boar taint genetic prediction using SENS. This would provide phenotypes for a reliable database for genetic evaluations, without following the same objectives as an online classification for carcass discrimination. This approach requires organizational changes compared to boar taint classification at slaughterhouses. Moreover, it is not realizable to routinely have ten trained assessors performing boar taint SENS assessment. Instead, the strategy could be to select a group of assessors showing the best SKA and ANON genetic correlations with SENS, combined with high heritability for the group. Applying this selection based on these combined estimated breeding values would optimize genetic progress for less boar taint risk. Combining SENS becomes also attractive hoping for more stable results over time, even if an assessor becomes unavailable.

As the data contained missing values, a combination of assessors was done a posteriori using already estimated (co)variances of the multivariate model. Therefore, in order to evaluate the optimal combination of several assessors, in the context of progeny-test evaluation, the genetic parameters of the mean and of different groups of SENSt were computed using covariance functions (e.g., Meyer and Hill, 1997) based on transformation of estimated covariance matrices. This method was applied to results from multivariate model using appropriate transformation matrices **T** of 3 × 12:


$$\begin{array}{l} T=\left[\begin{array}{ccccccccccccc} 1 & 0 & \ldots & 0 & \ldots \;0 & 1 & \ldots & 0 & \ldots \;0 & 0 & \ldots & c_{i} & \ldots \end{array}\right] \end{array}$$

Where $c_{i}$ is the contribution of assessor i to the mean $\left(c_{i}=\frac{1}{10}\right)$ or to the group of size n $\left(c_{i}=\frac{1}{n}\right)$, the coefficient being zero if the assessor is not a member of the group $\left(c_{i}=0\right)$. The transformed (co)variance matrices were then obtained as $R_{T}={TRT}^{\prime }$ and $G_{T}={TGT}^{\prime }$. Combinations were computed and sorted for heritability and genetic correlations with SKA and ANON. Genetic progress for SKA and ANON using the best combinations of assessors based on heritability or genetic correlations was compared using a correlated response equation (Hill, 2013).

## Results and Discussion

### Data transformation and statistics

First, we made a phenotypic study of SENS and assessor performances. Distribution of SENS into score classes for each assessor including means and SD by assessor are presented in Table 1. It revealed that assessors have their own perception of the assignment of taint scores, even though they were trained and selected for their ability to recognize SKA and ANON (Mörlein et al., 2016). Table 1 also contains SENSt that were calculated according to the sensory rating frequencies of each assessor. It was particularly informative for characterizing the usefulness of their scores as a boar taint phenotype and for introducing them into a genetic model. A Snell score transformation was applied to obtain an underlying continuous and noncategorical scale, and therefore suitable for linear models (Mujibi and Crews, 2009). Comparing SENS mean (1.13–2.63) to SENSt mean (−0.33 to −0.16) showed that SENSt centered more uniformly, correcting the individual scale usage by assessors. SD remained variable comparing SD ranges for SENS (1.45–1.91) and SENSt (1.12–1.57). Distributions of Snell scores are theoretically more comparable across assessors and differences in SENSt SD reflecting differences in the use of underlying normal scale. This was especially important for assessors 2, 4, 5, and 10 for which SENS class 1 was empty: SENSt differences between class 0 and 2 were corrected regarding this.

**Table 1.** Distribution of sensory scores (0–5) into score classes for each assessor (1–10), computed Snell score values for each class as used by the assessors; and the associated mean and SD

Assessor^1^	Score classes						Mean	SD
	0	1	2	3	4	5		
	Frequency							
1	0.43	0.10	0.12	0.17	0.13	0.05	1.60	1.68
2	0.48	0.00	0.08	0.19	0.19	0.06	1.80	1.84
3	0.28	0.02	0.17	0.25	0.21	0.07	2.30	1.67
4	0.35	0.00	0.07	0.23	0.20	0.15	2.38	1.91
5	0.52	0.00	0.05	0.22	0.12	0.09	1.67	1.85
6	0.13	0.17	0.14	0.20	0.22	0.14	2.63	1.63
7	0.59	0.08	0.11	0.10	0.06	0.06	1.13	1.59
8	0.28	0.09	0.21	0.19	0.17	0.06	2.05	1.62
9	0.21	0.18	0.26	0.19	0.11	0.05	1.95	1.45
10	0.52	0.00	0.22	0.14	0.07	0.05	1.39	1.60
All	0.38	0.06	0.14	0.19	0.15	0.08		
	Snell scores							
1	−1.65	−0.05	0.23	0.65	1.28	2.67	−0.29	1.31
2	−1.46	−0.05	0.05	0.40	1.10	2.59	−0.25	1.28
3	−2.35	−0.55	−0.30	0.25	1.03	2.51	−0.27	1.48
4	−2.00	−0.39	−0.29	0.09	0.71	2.12	−0.24	1.46
5	−1.31	0.05	0.13	0.28	0.58	2.39	−0.31	1.18
6	−3.45	−0.83	−0.34	0.09	0.71	2.16	−0.16	1.57
7	−1.06	0.33	0.61	0.97	1.36	2.58	−0.20	1.12
8	−2.35	−0.44	−0.05	0.47	1.15	2.59	−0.28	1.47
9	−2.73	−0.53	0.05	0.69	1.32	2.67	−0.26	1.49
10	−1.31	0.05	0.13	0.69	1.41	2.67	−0.33	1.16

^1^Number of animals scored equal to 1,016 except for assessors 4 and 5 who scored 856 animals.

As the primary objective of using trained assessors is the detection of boar taint without quantification of SKA and ANON, the boar taint perception can be characterized by performances of SENS compared to these major boar taint compound concentrations. Performances were evaluated using the “safe-box” approach, as presented by Mörlein et al. (2016). Therein, estimated consumer rejection thresholds for ANON (1.5 ppm) and SKA (0.2 ppm) were used. Moreover, SENS classes 2–5 were considered tainted (Mörlein et al., 2016). Results in Table 2 show ranges in sensitivity from 0.68 to 0.90, in specificity from 0.33 to 0.74, and in accuracy from 0.41 to 0.73. Our results are obviously close to those calculated and obtained by Mörlein et al. (2016) given near-identical data sets. The high sensitivity of assessor 9 (0.90), meant that in 9 out of 10 cases, this assessor detected that a sample was rated positive for boar taint when it was considered positive by chemical measurement too. Assessors 3 and 4 sensitivities were close to that of assessor 9. Assessor 7 was the least likely of the other assessors to detect (chemically) positive samples, but was the best for detecting negative samples, with a specificity of 0.74 and its measurements were the most reliable with an overall accuracy of 0.73. This one is followed by the performances of assessors 1 and 5. Assessors 3, 6, and 8 had the lowest accuracy and precision, and were not specific. Yet, the above findings on the sensitivity and specificity of assessors strongly depended on the chosen threshold levels of ANON and SKA. These in turn remain to be a subject of debate as they depend on the sensitivity of consumers, the type of product, its usage, and the chosen risk of consumer complaints. All these observations led to the conclusion that a single assessor would therefore be insufficient for reliable detection of boar taint with the required sensitivity and specificity. Moreover, although the goal of meat processors is to achieve a high true-positive rate (i.e., sensitivity) to avoid distributing tainted meat to consumers, breeding, for reduced boar taint occurrence, requires reliable phenotypes which implies high sensitivity as well as high specificity.

**Table 2.** Performances (sensitivity, specificity, precision, negative predictive value [NPV], and accuracy) for each assessor (1–10)

Assessor	Sensitivity^1^	Specificity^2^	Precision^3^	NPV^4^	Accuracy^5^
1	0.75	0.59	0.28	0.92	0.62
2	0.77	0.53	0.26	0.91	0.57
3	0.86	0.34	0.22	0.92	0.43
4	0.87	0.40	0.25	0.93	0.49
5	0.76	0.59	0.30	0.91	0.62
6	0.82	0.33	0.21	0.90	0.41
7	0.68	0.74	0.36	0.91	0.73
8	0.70	0.39	0.20	0.86	0.45
9	0.90	0.46	0.26	0.96	0.54
10	0.73	0.57	0.27	0.91	0.60

With TP as true-positives, FP as false-positives, TN as true-negatives, and FN as false-negatives.

^1^True positive rate = TP/(TP + FN).

^2^True negative rate = TN/(FP + TN).

^3^Positive predictive value = TP/(TP + FP).

^4^Negative predictive value = TN/(FN + TN).

^5^Accuracy = (TP + TN)/(TP + TN + FP + FN).

### Heritabilities and relationships between skatole and androstenone

The previous section demonstrated that some assessors seem to be less performant than others. However, it was not yet possible at this stage to determine which one was generating the best data for genetic selection against boar taint. One additional criterion for selecting assessors in this context could be the heritability of their SENSt trait. This is of importance to obtain a sustainable response to selection. Moreover, correlations to SKA and ANON are considered indicative of the compounds the assessor smells. Differences in perception could in fact be related to the presence of other odorous compounds and their interactions and therefore would be associated by the assessor to boar taint or could mask SKA and ANON olfactory strength. Variance components were estimated between SKAt, ANONt, and the 10 SENSt traits. First, SKAt and ANONt showed heritability estimates close to those found in the literature [i.e., 0.34–0.52 (Brinke et al., 2021)], with a heritability of 0.52 (SE = 0.09) for SKAt and 0.53 (SE = 0.08) for ANONt. The relationship between SKAt and ANONt was characterized by a phenotypic correlation of 0.38 (SE = 0.04) and a genetic correlation of 0.46 (SE = 0.11). The results obtained here confirmed that selecting pigs with low levels of major boar taint compounds is feasible, as Windig et al. (2012) concluded too.

### Heritabilities of SENSt traits

**Figure 1.**
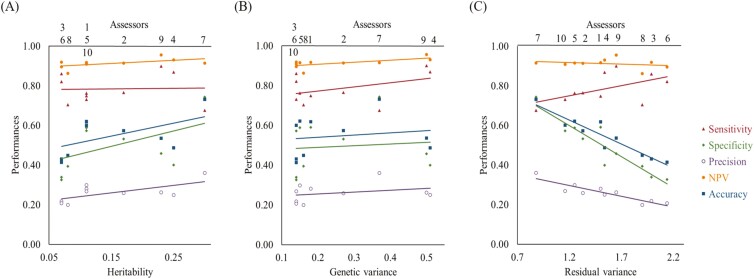
The relationship between performances and heritability (A), genetic variance (B), and residual variance (C) for the multivariate mixed model of all assessors (1–10).

Estimates of heritability for each SENSt are reported in Table 3. Results were low to moderate with a range between 0.07 (SE = 0.06) and 0.30 (SE = 0.08). This range was larger compared to previously reported values, i.e., 0.12–0.19 (Windig et al., 2012) and 0.11–0.14 (Mathur et al., 2013). These moderately high heritability for some SENSt, offered first promising prospects for the use of some SENSt traits in a genetic evaluation of boar taint. The highest heritability (0.30) was obtained for SENSt of assessor 7. Indeed, the latter has a high genetic variance and a remarkably low residual variance compared to the other assessors’ SENSt. This is notable because assessor 7 showed the lowest sensitivity (0.68) but the highest values in precision (0.36), and accuracy (0.73) compared to the other assessors. Genetic variances were even higher for SENSt of assessors 4 and 9, which explains their heritability values despite having rather high residual variance. The latter could reflect the attribution of a SENS of 0. As visible in Tables 1 and 3, the more an assessor attributed a SENS of 0, the lower the residual variance. At this step, it was necessary to study the connections between performances and genetic parameters through the computation of correlations (Figure 1). The relationship between genetic parameters and performances revealed that the residual variance reflects a linear inverse trend in specificity (r = −0.93), precision (r = −0.90), and accuracy (r = −0.94). Considering this, it must be highlighted that low residual variance is not equal to high heritability. For example, assessor 1 showed an accuracy of 0.62, being therefore, the second best based on this criterion. However, the heritability associated to its SENSt was only estimated to be 0.11, the third worst. These relationships between performances and genetic parameters revealed the need to verify residual and genetic variances of assessors before validating their power of discrimination on a genetic level. Overall, the results showed that selecting evaluators on the basis of phenotypic performance alone did not appear to be sufficient when considering the role of a given assessor, here being the recorded phenotype, in the context of breeding. This study showed that a strategy based on genetic analysis could be complementary for boar taint assessor validation.

**Table 3.** Heritabilities (h^2^ ± SE), (genetic ($\sigma_{u}^{2}$ ± SE) and residual ($\sigma_{e}^{2}$ ± SE)) variances of SENSt for each assessor (1–10), phenotypic (r_P_), genetic (r_G_) and residual (r_R_) correlations between assessors and skatole and androstenone obtained by the multivariate mixed model

Assessor	h² ± SE	$\sigma {\;}_{u}^{2}$ ± SE	$\sigma {\;}_{e}^{2}$ ± SE	Skatole			Androstenone		
				r_P_	r_G_	r_R_	r_P_	r_G_	r_R_
1	0.11 ± 0.06	0.18 ± 0.11	1.50 ± 0.11	0.36	0.84	0.24	0.21	0.52	0.13
2	0.17 ± 0.07	0.27 ± 0.12	1.33 ± 0.12	0.26	0.46	0.20	0.26	0.44	0.20
3	0.07 ± 0.06	0.14 ± 0.12	1.99 ± 0.14	0.24	0.81	0.13	0.12	0.29	0.10
4	0.25 ± 0.08	0.51 ± 0.17	1.54 ± 0.16	0.39	0.83	0.15	0.27	0.52	0.14
5	0.11 ± 0.07	0.15 ± 0.10	1.25 ± 0.11	0.38	0.79	0.30	0.30	0.61	0.24
6	0.07 ± 0.06	0.14 ± 0.14	2.14 ± 0.15	0.24	0.87	0.11	0.17	0.58	0.10
7	0.30 ± 0.08	0.37 ± 0.10	0.88 ± 0.09	0.43	0.72	0.25	0.29	0.50	0.17
8	0.08 ± 0.06	0.16 ± 0.12	1.90 ± 0.13	0.07	0.37	0.00	0.09	0.25	0.07
9	0.23 ± 0.07	0.50 ± 0.16	1.65 ± 0.14	0.45	0.86	0.25	0.27	0.53	0.13
10	0.11 ± 0.07	0.14 ± 0.09	1.16 ± 0.09	0.26	0.61	0.17	0.22	0.53	0.14

### Relationships of SENSt traits with SKAt and ANONt

The relationships between SENSt, SKAt, and ANONt were investigated by their genetic, phenotypic, and residual correlations presented in Table 3. For all SENSt, phenotypic correlations were low to moderate (from 0.07 to 0.45 for SKAt and from 0.09 to 0.30 for ANONt). They were higher for SKAt than ANONt as found in previous studies (Mathur et al., 2012; Mörlein et al., 2016) even though they were not calculated the same way. Genetic correlations tended to be stronger for SKAt (from 0.37 to 0.87) than for ANONt (from 0.25 to 0.61). These results showed that a relationship existed between the SENSt and the genetics of SKAt and ANONt. Moreover, the observed variability across these traits among assessors indicated that each assessor showed differences in genetic relationships to SKA and ANON. This confirmed the usefulness of defining SENS as phenotypes in the context of genetic selection.

Several studies indicated that the contribution of SKA to boar taint was generally higher than that of ANON, for which the sensitivity was lower (Weiler et al., 2000; Bekaert et al., 2011; Meier-Dinkel et al., 2013; Burgeon et al., 2021a). Finally, the residual correlations were null to moderate (from 0.00 to 0.30 for SKAt and from 0.07 to 0.24 for ANONt). A particular case was SENSt of assessor 6 with low heritability (0.07 ± 0.06) and high genetic correlation for SKA (0.87). This SENSt was genetically correlated to SKAt, but response to selection based on this SENS would be lower compared to others (because of low heritability). These results implied that the usefulness of the scores of this assessor was reduced in a progeny-test context. These results were explained by the high residual variance (2.14) found.

### Relationships between SENSt traits

Phenotypic (0.03–0.35) and genetic (0.11–0.89) correlations among SENSt are reported in Table 4. When genetic correlations were close to 1 (e.g. 0.89 between assessors 5 and 6 and 0.84 between assessors 8 and 10), assessors classified genetically related pigs in a similar way (i.e., through the same genetic background). SENS of assessor 8 seemed to be attributed in a totally different way (with phenotypic correlations of SENSt from 0.03 to 0.13 and genetic correlations of SENSt from 0.11 to 0.52 [excluding SENSt of assessor 10]) compared to the other SENSt. SENS of assessor 6 seemed to be attributed according to compounds correlated to boar taint commonly perceived by the other SENS with genetic correlations of SENSt ranging from 0.62 to 0.89. These results demonstrated that even though SENS attributions were probably similar due to the high genetic correlations between SENSt, the ranking of pigs and selection of (un)tainted pigs would be different from one assessor to another. Considering the range of phenotypic correlations, the estimates showed that perception among assessors varied. It reflects that olfactory perception varies between humans, including for boar taint (Windig et al., 2012), even if they were accustomed to recognize it. Moreover, conditions and training were the same for each assessor (Mörlein et al., 2016). As expected, they did not perceive the same compounds, since each olfactory system is unique. Olfactory perception is highly variable among individuals. This is due to various factors like environment and events. These include also genetics, and in particular to genes, which are at the root of this variability. Olfactory acuity depends on the presence of olfactory receptor genes and pseudogenes (Genva et al., 2019). If certain olfactory receptors develop, they are able to detect the compounds involved in boar taint. Considering this information and results, assessing the independence of the assessors’ measurement of SKA and ANON was investigated.

**Table 4.** Phenotypic (below diagonal) and genetic (above) correlations among the assessors (1–10) obtained by the multivariate mixed model

Assessor	Assessor									
	1	2	3	4	5	6	7	8	9	10
1		0.56	0.58	0.70	0.58	0.69	0.63	0.19	0.76	0.48
2	0.29		0.63	0.35	0.53	0.61	0.37	0.35	0.56	0.58
3	0.18	0.18		0.79	0.70	0.73	0.49	0.38	0.76	0.65
4	0.26	0.30	0.16		0.63	0.62	0.58	0.17	0.83	0.56
5	0.27	0.25	0.15	0.26		0.89	0.65	0.20	0.85	0.47
6	0.21	0.21	0.12	0.24	0.18		0.77	0.52	0.80	0.71
7	0.32	0.25	0.14	0.35	0.31	0.23		0.48	0.78	0.69
8	0.06	0.06	0.06	0.09	0.07	0.03	0.13		0.11	0.84
9	0.20	0.19	0.07	0.23	0.23	0.17	0.29	0.04		0.51
10	0.27	0.31	0.17	0.23	0.24	0.17	0.22	0.05	0.18	

### Recursive model

The multivariate mixed model usually used is not able to explain the direction of the relationships between traits. So, the relationships between SENS and SKA, and SENS and ANON were only partially estimated. The initial hypothesis was that SENS attribution by the sensory scores system was done because SKA and ANON were perceived by the assessors at a certain olfactive level. It should therefore be assumed that SENS had as origin SKA and ANON. To verify this hypothesis, a recursive model was applied. The recursive model was inferred from the genetic and residual (co)variance matrices of the multivariate model to study SENSt traits while holding the causal traits, that are, SKAt and ANONt, constant. The needed transformation matrix **Λ**, a 12 × 12 matrix of recursive model parameters, was estimated as


$$\begin{array}{l} \Lambda^{\prime }=\left[\begin{array}{llllllllllllllllllllllllllllllllllllllllllllllllllllllll} \;1 & \;0 & \;-1.18 & \;-0.77 & \;-0.70 & \;-0.65 & \;-1.27 & \;-0.58 & \;-0.92 & \;0.15 & \;-1.30 & \;-0.70\;0 & \;1 & \;-0.35 & \;-0.72 & \;-0.39 & \;-0.52 & \;-0.73 & \;-0.42 & \;-0.39 & \;-0.41 & \;-0.35 & \;-0.42\;0 & \;0 & \;1 & \;0 & \;0 & \;0 & \;0 & \;0 & \;0 & \;0 & \;0 & \;0\;... & \;\;... & \;... & \;... & \;... & \;... & \;... & \;... & \;... & \;... & \;... & \;...\;0 & \;0 & \;0 & \;0 & \;0 & \;0 & \;0 & \;0 & \;0 & \;0 & \;0 & \;1\; \end{array}\right]. \end{array}$$

Please note that the positive coefficient 0.15 between SKAt and SENSt of assessor 8 expressed a negative recursiveness, i.e., higher SENSt of assessor 8 values are caused by lower SKAt. It could reflect an ANON dominance to SKA and low SKA concentrations could imply ANON increasing perception. This can be also explained by the null SKA residual correlation compared to the residual correlation to ANON (Table 3).

Heritabilities, variances for SENSt traits, phenotypic, and genetic correlations of SENSt traits with SKAt and ANONt under the recursive model are presented in Table 5. Some SENSt traits remained heritable (0.04–0.19). For example, SENSt of assessor 7 (heritability of 0.19) was still measuring something moderately heritable, therefore considered potentially different from the linear perception of SKA and ANON. On the opposite, SENSt of assessor 8 heritability (0.08) was apparently unchanged because **Λ** was positive for SKA. Moreover, its genetic variances variation (1.19%) suggested that this assessor is not attributing SENS to SKA and ANON, since variances barely varied. This assessor probably smelled something else than SKA and ANON and identified it as taint. Moreover, the higher the genetic correlations of multivariate model with the causal traits were, the stronger the relative changes in heritability were, as explained by Varona and González-Recio (2023). For example, the SENSt of assessor 6 heritability switched from 0.07 (Table 3) to 0.02 (Table 5) probably because its genetic correlation of multivariate model (Table 3) with SKAt was high (0.87).

**Table 5.** Heritabilities (h²), (genetic [$\sigma_{u}^{2}$] and residual [$\sigma_{e}^{2}$]) variances of SENSt for each assessor (1–10), percentage of reduction of genetic variance between mixed and recursive models (**Δ**$\sigma_{u}^{2}$), phenotypic (r_P_) and genetic (r_G_) correlations between assessors and skatole and androstenone obtained by the recursive model

Assessor	h²	$\sigma {\;}_{u}^{2}$	Δ $\sigma {\;}_{u}^{2}$	$\sigma {\;}_{e}^{2}$	Skatole		Androstenone	
					r_P_	r_G_	r_P_	r_G_
1	0.04	0.05	71.08%	1.41	0.03	0.20	0.00	0.03
2	0.14	0.20	27.07%	1.25	−0.02	−0.06	−0.03	−0.10
3	0.04	0.07	48.97%	1.95	0.05	0.35	−0.03	−0.25
4	0.16	0.28	43.64%	1.50	0.20	0.71	0.09	0.31
5	0.05	0.06	62.21%	1.10	−0.05	−0.34	−0.06	−0.39
6	0.02	0.05	64.57%	2.10	0.08	0.67	0.03	0.24
7	0.19	0.19	46.89%	0.80	0.12	0.39	0.07	0.23
8	0.08	0.16	1.19%	1.90	0.07	0.34	0.01	0.03
9	0.11	0.19	62.30%	1.54	0.14	0.61	0.08	0.33
10	0.07	0.08	45.14%	1.11	0.02	0.08	0.01	0.07

As explained previously, these genetic parameters reflect (co)variance components among those traits while holding the causal traits, here SKAt and ANONt, constant. Residual correlations between causal traits and SENSt traits became zero. Phenotypic correlations (from −0.05 to 0.20 for SKAt; from −0.06 to 0.09 for ANONt) and genetic correlations (from −0.34 to 0.71 for SKAt; from −0.39 to 0.33 for ANONt) can be interpreted as relationships between SENSt and compounds correlated with SKA and ANON at constant levels of SKAt and ANONt. For example, SENSt of assessor 1 (with a genetic correlation of 0.20 for SKAt) described a moderate tendency to genetically group fats with an SKA-related compound. This correlation indicates probably a common genetic pathway with SKA and influences SENS assignment. On the opposite, SENSt of assessor 5 had negative genetic correlations with SKAt and ANONt (−0.34 and −0.39, respectively). A negative genetic correlation happened because the relationship between **Λ** and correlations follows a sigmoidal curve, and the direction of the recursive effect is reflected by this strong variation towards the negative values (Varona and González-Recio, 2023). Such a recursive effect relates to a strong initial dependence on SKA and ANON. Moreover, the proportion between variances dependent or not dependent on SKA and ANON (62.21%) indicated that most of the scores were attributed based on SKA and ANON. SENSt of assessor 5 had correlations with negatively correlated compound(s) to SKA and ANON.

Some hypotheses can be formulated to explain the results above about correlations and heritability. First, the study of Hansson et al. (1980) estimated that SKA and ANON explained only a part of the “boar taint” odor (50%). Second, fat tissue is a complex matrix made up of numerous compounds, including volatile organic compounds other than SKA and ANON. These compounds are released when the fat sample is heated and are therefore present along with SKA and ANON, which are relatively volatile compounds (Burgeon et al., 2021b, 2023; Rodrigues et al., 2024). Various studies have proposed that additional compounds, such as indole (contribution estimated at 7%), p-cresol and 4-ethylphenol, 4-phenyl-3-buten-2-one, 2-aminoacethophenone, and androstenols, may contribute to boar taint (Patterson, 1967; Hansson et al., 1980; Rius Solé and García Regueiro, 2001; Fischer et al., 2011, 2014; Gerlach et al., 2016; Mörlein et al., 2024). For example, indole is well known to be a boar taint compound with which SKA has phenotypic and genetic correlations of around 0.7 and 0.8 (Tajet et al., 2006; Windig et al., 2012). The presence of all previously cited compounds could potentially influence the detection of odor by the sensory evaluation. Conversely, when fat is heated to high temperatures, it may release other odorous compounds that could mask the presence of boar taint (i.e., saturation of the olfactory receptors) (Burgeon et al., 2021b). Finally, the interaction between SKA and ANON could contribute to another level of perception considering the potential non-linear relationship between these compounds that was described by Mörlein et al. (2016).

Table 6 contains genetic (from −0.42 to 0.83) and phenotypic (from −0.04 to 0.25) correlations among SENSt traits under a recursive model. All genetic correlations were reduced compared to the results of multivariate model as expected under the hypothesis that causal traits, here SKAt and ANONt, were responsible for these correlations. Some genetic correlations were still consistently high, such as the relationship between SENSt of assessors 8 and 10 (with a genetic correlation of 0.83). This could be interpreted as an indicator that these assessors, at fixed levels of SKAt and ANONt, assigned SENS in a repeatable and similar manner across pig families, i.e., genetically consistent. On the other hand, SENSt of assessors 5 and 1 (with a genetic correlation of −0.42) showed a completely opposite behavior in the same circumstances.

**Table 6.** Phenotypic (below the diagonal) and genetic (above) correlations of assessors (1–10) obtained with the recursive model

Assessor	Assessor									
	1	2	3	4	5	6	7	8	9	10
1		0.30	−0.12	0.10	−0.42	−0.09	0.07	−0.17	0.18	−0.17
2	0.21		0.47	−0.16	0.23	0.27	−0.04	0.18	0.19	0.35
3	0.11	0.13		0.49	0.20	0.37	−0.03	0.31	0.37	0.35
4	0.13	0.19	0.08		−0.38	0.16	0.18	0.03	0.61	0.06
5	0.13	0.15	0.06	0.09		0.18	−0.08	−0.27	0.12	−0.25
6	0.14	0.14	0.07	0.17	0.08		0.50	0.54	0.45	0.31
7	0.18	0.13	0.04	0.23	0.14	0.14		0.40	0.53	0.40
8	0.03	0.03	0.04	0.07	0.03	0.01	0.11		−0.16	0.83
9	0.04	0.06	−0.04	0.08	0.04	0.08	0.13	0.01		−0.08
10	0.18	0.25	0.11	0.13	0.13	0.11	0.11	0.03	0.06	

### Combining assessors for genetic selection

The last objective was to determine the optimal combination of assessors for reliably predicting genetically boar taint. This case is presented under specific conditions of recording relevant phenotypes for breeding programs, with the aim to select for reduced risk of the development of boar taint. Both high heritability, related to high direct response to selection, and genetic correlations with SKAt and ANONt, related to high indirect response to selection, are required. As a reference point, the heritability using the average of all assessors and their genetic correlations with SKAt and ANONt were computed (heritability of 0.32, genetic correlations of 0.91 with SKAt, and 0.61 with ANONt). A combination of three assessors was found to generate the highest heritability and the highest genetic correlations with SKAt and ANONt. Table 7 shows the three highest and three lowest combinations for heritability (A), for genetic correlations with SKAt (B), and with ANONt (C). All results of combinations are available in Supplementary Table S4. Using heritabilities, the three best combinations achieved heritabilities ranging from 0.34 to 0.40, higher than the average heritability of all SENSt. Genetic correlations for these combinations were at a high level for SKAt (from 0.89 to 0.91) and at a moderate to high level for ANONt (from 0.51 to 0.57). When using genetic correlations as a criterion to define sets of assessors, heritability highly decreased for SKAt (from 0.14 to 0.21) and ANONt (from 0.15 to 0.23). In practice, as direct genetic progress is largely depending on heritability which is an important parameter of the breeders equation (Hill, 2013), losing a large amount of heritability will decrease potential direct genetic progress for boar taint.

**Table 7.** The three best and worst sets of three assessors according to their heritability (A), genetic correlation with skatole (B), and androstenone (C) for the multivariate mixed model

Assessors groups			h²^1^	r_GSKAt_^1^	r_GANONt_^1^	r_PSKAt_^1^	r_PANONt_^1^
(A)							
4	7	9	0.40	0.89	0.57	0.40	0.26
1	4	9	0.34	0.91	0.57	0.38	0.24
3	4	9	0.34	0.90	0.51	0.38	0.22
…			…	…	…	…	…
1	5	8	0.13	0.90	0.62	0.23	0.16
3	5	8	0.13	0.83	0.49	0.21	0.13
1	3	8	0.12	0.88	0.47	0.22	0.12
(B)							
1	3	6	0.14	0.95	0.53	0.25	0.14
1	4	6	0.21	0.95	0.61	0.31	0.20
1	3	5	0.15	0.94	0.55	0.26	0.15
…			…	…	…	…	…
7	8	10	0.26	0.67	0.50	0.25	0.18
2	3	8	0.16	0.67	0.42	0.19	0.12
2	8	10	0.20	0.56	0.48	0.18	0.15
(C)							
1	5	10	0.15	0.91	0.67	0.25	0.19
5	6	10	0.16	0.85	0.64	0.24	0.18
4	5	10	0.23	0.90	0.64	0.31	0.22
…			…	…	…	…	…
3	6	8	0.13	0.81	0.45	0.21	0.12
2	3	8	0.16	0.67	0.42	0.19	0.12
3	8	10	0.16	0.68	0.41	0.20	0.12

^1^Heritability (h²), genetic correlations of skatole (r_GSKAt_) and androstenone (r_GANONt_), and phenotypic correlations of skatole (r_PSKAt_) and androstenone (r_PANONt_).

In the context of indirect correlated responses of SKA and ANON alternative optimization for genetic correlations showed slight increases of these genetic correlations (Table 7) but the loss due to lower heritability was not compensated for by the gain of the genetic response of maximum 7% for SKA and of maximum 18% for ANON for best sets of assessors. In terms of genetic gain, using the best set of assessors based on heritability compared to the best sets based on genetic correlations, the genetic gain estimated would approximately be reduced to 63% for SKA and 72% for ANON. Based on these considerations, the trio of assessors (4, 7, and 9) would represent the best combination. This combination would allow us to associate a more important part of the population variability to genetic predictions, and as a consequence to select more efficiently and to reduce the risk of boar taint development by three different assessments. Finally, this methodology could be applied to find the best combination of assessors in a practical context as described in the beginning of this section. Indeed, phenotyping in a genetic context has as objective the provision of a relevant database for genetic evaluation, which can be considered different from carcass classification. Moreover, it implies to consider back fat sampling and storage in order to carry out post-slaughtering sensory analyses of boar taint. From a practical and potentially financial point of view, this would mean no longer having to use the costly and time-consuming SKA and ANON measurements, except for eventual controls, and replacing these analyses with sensory analyses by several trained assessors. This situation requires an employee available for sampling, storage, and organizing traceability (Burgeon et al., 2021a). Conversely to online assessments, lab sensory evaluations (typically) are carried out under controlled conditions. In the context of routine testing, 10 trained assessors will obviously not be available. Therefore selecting a group of trained assessors whose SENS assessments were tested for heritability and correlations with SKA and ANON, was proposed. Considering that they perceive and assess boar taint, SKA and ANON, differently, when combined, they contribute better than considered as individual trait. Using several assessors provides stability in the hypothetical situation where an assessor is unavailable or stops working, he/she could be replaced by another. Finally, the main costs would concern the training of assessors, the salaries of assessors, and additional employee(s) during the slaughtering and implementation of the routine organization (Burgeon et al., 2021a). Yet, for the acquisition of high-quality phenotypes, for example in the context of genomic selection, covering these costs could be defended. Obviously, the cost-benefit ratio depends on many factors such as the cost of the SKA and ANON analyses, and the salaries of employees and assessors.

## Conclusions

In our study, we investigated the use of sensory evaluation phenotypes for characterizing boar taint in the context of animal breeding. We compared individual assessors and characterized trained assessors by their performances. We highlighted that distributions were highly heterogeneous between score assignments, which reflect the different sensory perceptions and decision-making behavior of assessors, even though they were trained. Moreover, the performance study revealed the need for reliable phenotypes which implies high sensitivity as well as high specificity.

By estimating variance components, the results revealed that some assessors obtained heritable scores as shown by moderate heritabilities, which validated that their scores are usable for genetic evaluation purposes. One might conclude that this type of analysis should allow to develop a strategy to select assessors in a progeny-test context. Moreover, according to obtained genetic correlations, genetic selection using sensory ratings of boar taint perception will decrease SKA, and to a lesser extent, ANON. Performance relationship to heritability revealed that we cannot base the use of one or more assessors solely on their phenotypic performances, for a reliable selection against boar taint.

Through the use of a posteriori recursive model, we found that, after correcting for SKA and ANON, some assessors had high genetic correlations which means a similar behavior, implying that there are potentially other substances interfering with the perception of ANON and SKA and thus affecting the performances of these assessors. A better understanding of volatile organic compounds leading to boar taint and their interaction, including SKA and ANON interaction, could help sensory training. Moreover, to perform this study, a linear approach has been used that implies the assumption that if boar taint is more pronounced (i.e., SENS is high), the concentrations of compounds are higher. However, the interaction between SKA and ANON has been demonstrated as being non-linear by other studies. This aspect will require further research in the future as a better understanding of boar taint phenotypes would help to improve genetic modeling.

In a breeding perspective context, results also showed that, in order to assess boar taint with a high degree of certainty, it would be important to use a combination of multiple assessors as linear combinations of assessors were found to be advantageous over single assessors. This methodology would facilitate genetic selection, that is, reducing boar taint risk, and therefore consumer dissatisfaction.

In a broader context, the proposed genetic investigation strategy, based on the use of recursive models, could allow disentangling sensory evaluations from causal traits also in other contexts. As shown in this study, genetic investigations of sensory evaluations could complement performance assessment to validate attributions of scores by trained assessors.

## Supplementary Material

skae389_suppl_Supplementary_Materials

## Data Availability

The datasets used and analyzed during the current study are available from Dr. Ernst Tholen (etho@itw.uni-bonn.de) on reasonable request and with the permission of the involved breeding organizations.

## Abbreviations

AI-REML: average information restricted estimate maximum likelihood
ANON: androstenone
ANONt: log-transformed androstenone
EM-REML: expectation maximization restricted estimate maximum likelihood
F1: crossbred commercial sows
FN: false-negative
FP: false-positive
NPV: negative predictive value
Pi: Pietrain
REML: restricted estimate maximum likelihood
SENS: sensory scores obtained by trained sensory assessors
SENSt: Snell transformed sensory scores obtained by trained sensory assessors
SIDA-HS-SPME-GC-MS: stable isotope dilution-headspace solid-phase microextraction-gas chromatography-mass spectrometry
SKA: skatole
SKAt: log-transformed skatole
TN: true-negative
TP: true-positive

## References

[CIT0001] Ausschuss für Leistungsprüfung und Zuchtwertschätzung (ALZ) beim Schwein des Zentralverbandes der Deutschen Schweineproduktion (ZDS). 2007. Richtlinie für die Stationsprüfung auf Mastleistung, Schlachtkörperwert und Fleischbeschaffenheit beim Schwein. Bonn (Germany): Zentralverband der Deutschen Schweineproduktion e.V. (ZDS).

[CIT0002] Bekaert, K. M., F. A. M. Tuyttens, L. Duchateau, H. F. De Brabander, M. Aluwé, S. Millet, F. Vandendriessche, and L. Vanhaecke. 2011. The sensitivity of Flemish citizens to androstenone: influence of gender, age, location and smoking habits. Meat Sci. 88:548–552. doi: https://doi.org/10.1016/j.meatsci.2011.02.010 21396787

[CIT0003] Bonneau, M., and U. Weiler. 2019. Pros and cons of alternatives to piglet castration: welfare, boar taint, and other meat quality traits. Animals 9:884. doi: https://doi.org/10.3390/ani9110884 31671665 PMC6912452

[CIT0004] Borrisser-Pairó, F., N. Panella-Riera, M. Gil, Z. Kallas, M. B. Linares, M. Egea, M. D. Garrido, and M. A. Oliver. 2017. Consumers’ sensitivity to androstenone and the evaluation of different cooking methods to mask boar taint. Meat Sci. 123:198–204. doi: https://doi.org/10.1016/j.meatsci.2016.10.006 27756017

[CIT0005] Brinke, I., C. Große-Brinkhaus, K. Roth, M. J. Pröll-Cornelissen, S. Klein, K. Schellander, and E. Tholen. 2021. Endocrine fertility parameters—Genomic background and their genetic relationship to boar taint in German Landrace and Large White. Animals 11:231. doi: https://doi.org/10.3390/ani11010231 33477702 PMC7831948

[CIT0006] Brinke, I., C. Groβe-Brinkhaus, K. Roth, M. J. Pröll-Cornelissen, I. Schiefler, and E. Tholen. 2022. Meta-analyses for boar taint compounds in two purebred maternal lines and Piétrain-sired crosses. Proceedings of 12th World Congress on Genetics Applied to Livestock Production (WCGALP). Wageningen (The Netherlands): Wageningen Academic Publishers; p. 3302–3305. doi:10.3920/978-90-8686-940-4_802

[CIT0007] Burgeon, C., M. Debliquy, D. Lahem, J. Rodriguez, A. Ly, and M. -L. Fauconnier. 2021a. Past, present, and future trends in boar taint detection. Trends Food Sci. Technol. 112:283–297. doi: https://doi.org/10.1016/j.tifs.2021.04.007

[CIT0008] Burgeon, C., A. Markey, M. Debliquy, D. Lahem, J. Rodriguez, A. Ly, and M. -L. Fauconnier. 2021b. Comprehensive SPME-GC-MS analysis of VOC profiles obtained following high-temperature heating of pork back fat with varying boar taint intensities. Foods 10:1311. doi: https://doi.org/10.3390/foods10061311 34200407 PMC8227496

[CIT0009] Burgeon, C., A. Markey, Y. Brostaux, and M. -L. Fauconnier. 2023. Modifying headspace sampling environment improves detection of boar taint compounds in pork fat samples. Chemosensors 11:551. doi: https://doi.org/10.3390/chemosensors11110551

[CIT0010] Dempster, A. P., N. M. Laird, and D. B. Rubin. 1977. Maximum likelihood from incomplete data via the *EM* algorithm. J. R. Stat. Soc. Ser. B: Stat. Method. 39:1–22. doi: https://doi.org/10.1111/j.2517-6161.1977.tb01600.x

[CIT0011] Deutscher Bundestag. 2018. Entwurf eines Vierten Gesetzes zur Änderung des Tierschutzgesetzes. Drucksache 19/5522. Available from https://dserver.bundestag.de/btd/19/055/1905522.pdf

[CIT0012] Duarte, D. A. S., M. Schroyen, R. R. Mota, S. Vanderick, and N. Gengler. 2021. Recent genetic advances on boar taint reduction as an alternative to castration: a review. J. Appl. Genet. 62:137–150. doi: https://doi.org/10.1007/s13353-020-00598-w 33405214 PMC7822767

[CIT0013] Eaton, J. W., D. Bateman, S. Hauberg, and R. Wehbring. 2020. GNU Octave version 6.1.0 manual: a high-level interactive language for numerical computations. Available from https://www.gnu.org/software/octave/doc/v6.3.0/

[CIT0014] Fischer, J., P. W. Elsinghorst, M. Bücking, E. Tholen, B. Petersen, and M. Wüst. 2011. Development of a candidate reference method for the simultaneous quantitation of the boar taint compounds androstenone, 3α-androstenol, 3β-androstenol, skatole, and indole in pig fat by means of stable isotope dilution analysis–headspace solid-phase microextraction–gas chromatography/mass spectrometry. Anal. Chem. 83:6785–6791. doi: https://doi.org/10.1021/ac201465q 21800819

[CIT0015] Fischer, J., C. Gerlach, L. Meier-Dinkel, P. W. Elsinghorst, P. Boeker, H. -G. Schmarr, and M. Wüst. 2014. 2-Aminoacetophenone—a hepatic skatole metabolite as a potential contributor to boar taint. Food Res. Int. 62:35–42. doi: https://doi.org/10.1016/j.foodres.2014.02.045

[CIT0016] Font-i-Furnols, M., R. Martín-Bernal, M. Aluwé, M. Bonneau, J. -E. Haugen, D. Mörlein, J. Mörlein, N. Panella-Riera, and M. Škrlep. 2020. Feasibility of on/at line methods to determine boar taint and boar taint compounds: an overview. Animals 10:1886. doi: https://doi.org/10.3390/ani10101886 33076492 PMC7602555

[CIT0017] Genva, M., T. Kenne Kemene, M. Deleu, L. Lins, and M. -L. Fauconnier. 2019. is it possible to predict the odor of a molecule on the basis of its structure? Int. J. Mol. Sci. 20:3018. doi: https://doi.org/10.3390/ijms20123018 31226833 PMC6627536

[CIT0018] Gerlach, C., P. W. Elsinghorst, H. -G. Schmarr, and M. Wüst. 2016. 2-Aminoacetophenone is the main volatile phase I skatole metabolite in Pietrain × Baden-Württemberg hybrid type boars. J. Agric. Food Chem. 64:1158–1163. doi: https://doi.org/10.1021/acs.jafc.5b05729 26804051

[CIT0019] Gianola, D., and D. Sorensen. 2004. Quantitative genetic models for describing simultaneous and recursive relationships between phenotypes. Genetics 167:1407–1424. doi: https://doi.org/10.1534/genetics.103.025734 15280252 PMC1470962

[CIT0020] Gilmour, A. R., R. Thompson, and B. R. Cullis. 1995. Average information REML: an efficient algorithm for variance parameter estimation in linear mixed models. Biometrics 51:1440–1450. doi: https://doi.org/10.2307/2533274

[CIT0021] Haberland, A. M., H. Luther, A. Hofer, E. Tholen, H. Simianer, B. Lind, and C. Baes. 2014. Efficiency of different selection strategies against boar taint in pigs. Animal 8:11–19. doi: https://doi.org/10.1017/S1751731113001857 24176119

[CIT0022] Hansson, K. -E., K. Lundström, S. Fjelkner-Modig, and J. Persson. 1980. The importance of androstenone and skatole for boar taint. Swed. J. Agric. Res. 10:167–173.

[CIT0023] Heyrman, E., S. Janssens, N. Buys, L. Vanhaecke, S. Millet, F. A. M. Tuyttens, J. Wauters, and M. Aluwé. 2020. Developing and understanding olfactory evaluation of boar taint. Animals 10:1684. doi: https://doi.org/10.3390/ani10091684 32957708 PMC7552758

[CIT0024] Hill, W. G. 2013. Artificial selection. In: Maloy, S., and K. Hughes, editors. Brenner’s encyclopedia of genetics. San Diego (CA): Academic Press; p. 200–203. doi:10.1016/B978-0-12-374984-0.00096-6

[CIT0025] Lundström, K., K. R. Matthews, and J. -E. Haugen. 2009. Pig meat quality from entire males. Animal 3:1497–1507. doi: https://doi.org/10.1017/S1751731109990693 22444983

[CIT0026] Mathur, P. K., J. ten Napel, S. Bloemhof, L. Heres, E. F. Knol, and H. A. Mulder. 2012. A human nose scoring system for boar taint and its relationship with androstenone and skatole. Meat Sci. 91:414–422. doi: https://doi.org/10.1016/j.meatsci.2012.02.025 22436660

[CIT0027] Mathur, P. K., J. Napel, R. E. Crump, H. A. Mulder, and E. F. Knol. 2013. Genetic relationship between boar taint compounds, human nose scores, and reproduction traits in pigs. J. Anim. Sci. 91:4080–4089. doi: https://doi.org/10.2527/jas.2013-6478 23825333

[CIT0028] Meier-Dinkel, L., A. R. Sharifi, E. Tholen, L. Frieden, M. Bücking, M. Wicke, and D. Mörlein. 2013. Sensory evaluation of boar loins: trained assessors’ olfactory acuity affects the perception of boar taint compounds. Meat Sci. 94:19–26. doi: https://doi.org/10.1016/j.meatsci.2012.12.009 23357575

[CIT0029] Meier-Dinkel, L., J. Gertheiss, S. Müller, R. Wesoly, and D. Mörlein. 2015. Evaluating the performance of sensory quality control: the case of boar taint. Meat Sci. 100:73–84. doi: https://doi.org/10.1016/j.meatsci.2014.09.013 25310880

[CIT0030] Meyer, K., and W. G. Hill. 1997. Estimation of genetic and phenotypic covariance functions for longitudinal or ‘repeated’ records by restricted maximum likelihood. Livest. Prod. Sci. 47:185–200. doi: https://doi.org/10.1016/s0301-6226(96)01414-5

[CIT0031] Meyer, K., and D. Houle. 2013. Sampling based approximation of confidence intervals for functions of genetic covariance matrices. Proc. Assoc. Advmt. Anim. Breed. Genet. 20:523–526. Available from http://www.aaabg.org/aaabghome/AAABG20- papers/meyer20523.pdf

[CIT0032] Ministère de l’agriculture et de l’alimentation. 2021. Arrêté du 17 novembre 2021 modifiant l’arrêté du 24 février 2020 modifiant l’arrêté du 16 janvier 2003 établissant les normes minimales relatives à la protection des porcs. NOR : AGRT2134285A. Texte n°34. Available from https://www.legifrance.gouv.fr/jorf/id/JORFTEXT000044340570

[CIT0033] Misztal, I., S. Tsuruta, D. A. L. Lourenco, Y. Masuda, I. Aguilar, A. Legarra, and Z. Vitezica. 2014. Manual for BLUPF90 family programs. Georgia: University of Georgia. Available from https://nce.ads.uga.edu/html/projects/programs/docs/blupf90_all8.pdf

[CIT0034] Mörlein, D., J. Trautmann, J. Gertheiss, L. Meier-Dinkel, J. Fischer, H. -J. Eynck, L. Heres, C. Looft, and E. Tholen. 2016. Interaction of skatole and androstenone in the olfactory perception of boar taint. J. Agric. Food Chem. 64:4556–4565. doi: https://doi.org/10.1021/acs.jafc.6b00355 27180946

[CIT0035] Mörlein, D., J. Mörlein, C. Gerlach, M. Strack, B. Kranz, and D. A. Brüggemann. 2024. An overlooked compound contributing to boar taint and consumer rejection of meat products: 2-aminoacetophenone. Meat Sci. 213:109497. doi: https://doi.org/10.1016/j.meatsci.2024.109497 38508078

[CIT0036] Mujibi, F. D. N., and D. H. Crews. 2009. Genetic parameters for calving ease, gestation length, and birth weight in Charolais cattle. J. Anim. Sci. 87:2759–2766. doi: https://doi.org/10.2527/jas.2008-1141 19465493

[CIT0037] Parois, S. P., A. Prunier, M. J. Mercat, E. Merlot, and C. Larzul. 2015. Genetic relationships between measures of sexual development, boar taint, health, and aggressiveness in pigs. J. Anim. Sci. 93:3749–3758. doi: https://doi.org/10.2527/jas.2014-8290 26440153

[CIT0038] Patterson, R. L. S. 1967. A possible contribution of phenolic components to boar odour. J. Sci. Food Agric. 18:8–10. doi: https://doi.org/10.1002/jsfa.2740180103

[CIT0039] R Core Team. 2022. R: a language and environment for statistical computing. Vienna, Austria: R Foundation for Statistical Computing. https://www.R-project.org/.

[CIT0040] Rius Solé, M. A., and J. A. García Regueiro. 2001. Role of 4-Phenyl-3-buten-2-one in boar taint: identification of new compounds related to sensorial descriptors in pig fat. J. Agric. Food Chem. 49:5303–5309. doi: https://doi.org/10.1021/jf010482d 11714320

[CIT0041] Robic, A., T. Faraut, and A. Prunier. 2014. Pathways and genes involved in steroid hormone metabolism in male pigs: a review and update. J. Steroid Biochem. Mol. Biol. 140:44–55. doi: https://doi.org/10.1016/j.jsbmb.2013.11.001 24239507

[CIT0042] Rodrigues, A., T. Massenet, L. M. Dubois, A. -C. Huet, A. Markey, J. Wavreille, N. Gengler, P. -H. Stefanuto, and J. -F. Focant. 2024. Development and validation of a classification model for boar taint detection in pork fat samples. Food Chem. 443:138572. doi: https://doi.org/10.1016/j.foodchem.2024.138572 38295570

[CIT0043] Snell, E. J. 1964. A Scaling procedure for ordered categorical data. Biometrics 20:592–607. doi: https://doi.org/10.2307/2528498

[CIT0044] Squires, E. J., C. Bone, and J. Cameron. 2020. Pork production with entire males: directions for control of boar taint. Animals 10:1665. doi: https://doi.org/10.3390/ani10091665 32947846 PMC7552340

[CIT0045] Tajet, H., O. Andresen, and T. Meuwissen. 2006. Estimation of genetic parameters of boar taint; skatole and androstenone and their correlations with sexual maturation. Acta Vet. Scand. 48:S9. doi: https://doi.org/10.1186/1751-0147-48-S1-S9

[CIT0046] Varona, L., and O. González-Recio. 2023. Invited review: recursive models in animal breeding: interpretation, limitations, and extensions. J. Dairy Sci. 106:2198–2212. doi: https://doi.org/10.3168/jds.2022-22578 36870846

[CIT0047] Verplanken, K., S. Stead, R. Jandova, C. Van Poucke, J. Claereboudt, J. Vanden Bussche, S. De Saeger, Z. Takats, J. Wauters, and L. Vanhaecke. 2017. Rapid evaporative ionization mass spectrometry for high-throughput screening in food analysis: the case of boar taint. Talanta 169:30–36. doi: https://doi.org/10.1016/j.talanta.2017.03.056 28411818

[CIT0048] Weiler, U., M. F. i Furnols, K. Fischer, H. Kemmer, M. A. Oliver, M. Gispert, A. Dobrowolski, and R. Claus. 2000. Influence of differences in sensitivity of Spanish and German consumers to perceive androstenone on the acceptance of boar meat differing in skatole and androstenone concentrations. Meat Sci. 54:297–304. doi: https://doi.org/10.1016/S0309-1740(99)00106-0 22060699

[CIT0049] Wesoly, R., and U. Weiler. 2012. Nutritional influences on skatole formation and skatole metabolism in the pig. Animals 2:221–242. doi: https://doi.org/10.3390/ani2020221 26486918 PMC4494329

[CIT0050] Windig, J. J., H. A. Mulder, J. ten Napel, E. F. Knol, P. K. Mathur, and R. E. Crump. 2012. Genetic parameters for androstenone, skatole, indole, and human nose scores as measures of boar taint and their relationship with finishing traits. J. Anim. Sci. 90:2120–2129. doi: https://doi.org/10.2527/jas.2011-4700 22247111

[CIT0051] Zamaratskaia, G., and E. J. Squires. 2009. Biochemical, nutritional and genetic effects on boar taint in entire male pigs. Animal 3:1508–1521. doi: https://doi.org/10.1017/S1751731108003674 22444984

