# Supplementary material for: Genetic investigations into the use of sensory evaluation: the case of boar taint discrimination in Pietrain sired crossbreds
Source: J Anim Sci. 2024 Dec 24;103:skae389. doi: 10.1093/jas/skae389 (PMC11747698; doi:10.1093/jas/skae389)
Supplement: skae389_suppl_Supplementary_Materials [file skae389_suppl_supplementary_materials.docx]

### Supplementary data

**Supplementary material 1.** Descriptive statistics of skatole, androstenone and transformed skatole and androstenone by log (base 10).

|  | Mean | SD | Min | Max |
| --- | --- | --- | --- | --- |
| Skatole (ppb) | 116.95 | 132.49 | 13 | 1453 |
| Androstenone (ppb) | 651.1 | 782.43 | 38 | 6412 |
| Log_10_(Skatole) | 1.92 | 0.33 | 1.11 | 3.16 |
| Log_10_(Androstenone) | 2.62 | 0.4 | 1.59 | 3.81 |

**Supplementary material 2.** Fixed solutions (x10^-3^), SE (x10^-3^) for regression coefficients on weight and age and approximate t statistics

|  | | | **Weight** | | | **Age** | | |
| --- | --- | --- | --- | --- | --- | --- | --- | --- |
|  | **Solutions** | **SE** | | **Approximative t-value** | **Solutions** | | **SE** | **Approximative t-value** |
| **Skatole** | -0.40 | 2.20 | | 0.18 | -0.93 | | 1.09 | 0.85 |
| **Androstenone** | 0.30 | 2.47 | | 0.12 | 1.19 | | 1.23 | 0.97 |
| **Assessor 1** | -18.51 | 9.29 | | 1.99 | 2.86 | | 4.24 | 0.67 |
| **Assessor 2** | -10.36 | 9.04 | | 1.15 | 7.52 | | 4.20 | 1.79 |
| **Assessor 3** | 0.38 | 10.50 | | 0.04 | 3.41 | | 4.75 | 0.72 |
| **Assessor 4** | -31.21 | 11.20 | | 2.79 | 8.20 | | 5.28 | 1.55 |
| **Assessor 5** | -18.73 | 9.36 | | 2.00 | -0.03 | | 4.32 | 0.01 |
| **Assessor 6** | -22.84 | 10.84 | | 2.11 | -8.78 | | 4.90 | 1.79 |
| **Assessor 7** | -22.13 | 7.82 | | 2.83 | 1.40 | | 3.72 | 0.38 |
| **Assessor 8** | 1.90 | 10.34 | | 0.18 | -4.02 | | 4.70 | 0.86 |
| **Assessor 9** | -2.41 | 10.34 | | 0.23 | 3.26 | | 4.84 | 0.67 |
| **Assessor 10** | -14.03 | 8.17 | | 1.72 | 9.50 | | 3.74 | 2.54 |

**Supplementary material 3.** Standard errors for genetic covariances (a) and residual covariances (b).

| **(a)** | **Skatole** | **Androstenone** | **Assessors** | | | | | | | | | |
| --- | --- | --- | --- | --- | --- | --- | --- | --- | --- | --- | --- | --- |
|  |  |  | **1** | **2** | **3** | **4** | **5** | **6** | **7** | **8** | **9** | **10** |
| **Skatole** | 0.01 | 0.01 | 0.03 | 0.03 | 0.03 | 0.03 | 0.03 | 0.03 | 0.03 | 0.03 | 0.03 | 0.02 |
| **Androstenone** | 0.01 | 0.01 | 0.03 | 0.03 | 0.03 | 0.04 | 0.03 | 0.03 | 0.03 | 0.03 | 0.03 | 0.02 |
| **1** | 0.03 | 0.03 | 0.11 | 0.09 | 0.09 | 0.10 | 0.08 | 0.09 | 0.08 | 0.08 | 0.10 | 0.07 |
| **2** | 0.03 | 0.03 | 0.09 | 0.12 | 0.09 | 0.11 | 0.08 | 0.10 | 0.08 | 0.09 | 0.10 | 0.08 |
| **3** | 0.03 | 0.03 | 0.09 | 0.09 | 0.12 | 0.11 | 0.08 | 0.10 | 0.08 | 0.09 | 0.10 | 0.08 |
| **4** | 0.03 | 0.04 | 0.10 | 0.11 | 0.11 | 0.17 | 0.10 | 0.12 | 0.10 | 0.11 | 0.13 | 0.09 |
| **5** | 0.03 | 0.03 | 0.08 | 0.08 | 0.08 | 0.10 | 0.10 | 0.09 | 0.08 | 0.08 | 0.10 | 0.07 |
| **6** | 0.03 | 0.03 | 0.09 | 0.10 | 0.10 | 0.12 | 0.09 | 0.14 | 0.09 | 0.09 | 0.11 | 0.08 |
| **7** | 0.03 | 0.03 | 0.08 | 0.08 | 0.08 | 0.10 | 0.08 | 0.09 | 0.10 | 0.08 | 0.10 | 0.07 |
| **8** | 0.03 | 0.03 | 0.08 | 0.09 | 0.09 | 0.11 | 0.08 | 0.09 | 0.08 | 0.12 | 0.10 | 0.07 |
| **9** | 0.03 | 0.03 | 0.10 | 0.10 | 0.10 | 0.13 | 0.10 | 0.11 | 0.10 | 0.10 | 0.16 | 0.08 |
| **10** | 0.02 | 0.02 | 0.07 | 0.08 | 0.08 | 0.09 | 0.07 | 0.08 | 0.07 | 0.07 | 0.08 | 0.09 |
| **(b)** | **Skatole** | **Androstenone** | **Assessors** | | | | | | | | | |
|  |  |  | **1** | **2** | **3** | **4** | **5** | **6** | **7** | **8** | **9** | **10** |
| **Skatole** | 0.01 | 0.01 | 0.02 | 0.02 | 0.02 | 0.03 | 0.02 | 0.03 | 0.02 | 0.02 | 0.03 | 0.02 |
| **Androstenone** | 0.01 | 0.01 | 0.02 | 0.02 | 0.03 | 0.03 | 0.02 | 0.03 | 0.02 | 0.03 | 0.03 | 0.02 |
| **1** | 0.02 | 0.02 | 0.11 | 0.09 | 0.09 | 0.10 | 0.08 | 0.10 | 0.08 | 0.09 | 0.09 | 0.08 |
| **2** | 0.02 | 0.02 | 0.09 | 0.12 | 0.09 | 0.10 | 0.08 | 0.10 | 0.07 | 0.09 | 0.09 | 0.08 |
| **3** | 0.02 | 0.03 | 0.09 | 0.09 | 0.14 | 0.11 | 0.09 | 0.11 | 0.08 | 0.10 | 0.10 | 0.08 |
| **4** | 0.03 | 0.03 | 0.10 | 0.10 | 0.11 | 0.16 | 0.10 | 0.12 | 0.09 | 0.10 | 0.11 | 0.09 |
| **5** | 0.02 | 0.02 | 0.08 | 0.08 | 0.09 | 0.10 | 0.11 | 0.09 | 0.07 | 0.09 | 0.09 | 0.07 |
| **6** | 0.03 | 0.03 | 0.10 | 0.10 | 0.11 | 0.12 | 0.09 | 0.15 | 0.09 | 0.10 | 0.11 | 0.09 |
| **7** | 0.02 | 0.02 | 0.08 | 0.07 | 0.08 | 0.09 | 0.07 | 0.09 | 0.09 | 0.08 | 0.09 | 0.07 |
| **8** | 0.02 | 0.03 | 0.09 | 0.09 | 0.10 | 0.10 | 0.09 | 0.10 | 0.08 | 0.13 | 0.10 | 0.08 |
| **9** | 0.03 | 0.03 | 0.09 | 0.09 | 0.10 | 0.11 | 0.09 | 0.11 | 0.09 | 0.10 | 0.14 | 0.08 |
| **10** | 0.02 | 0.02 | 0.08 | 0.08 | 0.08 | 0.09 | 0.07 | 0.09 | 0.07 | 0.08 | 0.08 | 0.09 |

**Supplementary material 4.** Heritability (h²), genetic correlations with skatole (r_GSKAt_) and androstenone (r_GANDt_), and phenotypic correlations with skatole (r_PSKAt_) and androstenone (r_PANDt_) of sets of three assessors for the multivariate mixed model

| **Assessors groups** | | | **h²** | **r_GSKAt_** | **r_GANDt_** | **r_PSKAt_** | **r_PANDt_** |
| --- | --- | --- | --- | --- | --- | --- | --- |
| 4 | 7 | 9 | 0.40 | 0.89 | 0.57 | 0.40 | 0.26 |
| 4 | 5 | 9 | 0.34 | 0.90 | 0.59 | 0.38 | 0.25 |
| 4 | 9 | 10 | 0.33 | 0.90 | 0.59 | 0.37 | 0.25 |
| 7 | 9 | 10 | 0.33 | 0.85 | 0.58 | 0.35 | 0.24 |
| 5 | 7 | 9 | 0.33 | 0.86 | 0.58 | 0.36 | 0.24 |
| 2 | 7 | 9 | 0.33 | 0.82 | 0.58 | 0.34 | 0.24 |
| 2 | 4 | 9 | 0.32 | 0.86 | 0.59 | 0.35 | 0.24 |
| 4 | 5 | 7 | 0.29 | 0.90 | 0.62 | 0.35 | 0.24 |
| 1 | 4 | 9 | 0.34 | 0.91 | 0.57 | 0.38 | 0.24 |
| 4 | 7 | 10 | 0.30 | 0.86 | 0.60 | 0.34 | 0.24 |
| 1 | 7 | 9 | 0.33 | 0.89 | 0.57 | 0.36 | 0.24 |
| 2 | 4 | 7 | 0.27 | 0.87 | 0.62 | 0.33 | 0.23 |
| 4 | 6 | 9 | 0.30 | 0.92 | 0.59 | 0.36 | 0.23 |
| 1 | 4 | 7 | 0.29 | 0.91 | 0.59 | 0.35 | 0.23 |
| 6 | 7 | 9 | 0.30 | 0.88 | 0.57 | 0.35 | 0.23 |
| 5 | 9 | 10 | 0.24 | 0.89 | 0.63 | 0.31 | 0.22 |
| 4 | 6 | 7 | 0.26 | 0.92 | 0.60 | 0.34 | 0.22 |
| 2 | 5 | 9 | 0.27 | 0.81 | 0.59 | 0.30 | 0.22 |
| 4 | 5 | 10 | 0.23 | 0.90 | 0.64 | 0.31 | 0.22 |
| 4 | 8 | 9 | 0.28 | 0.92 | 0.58 | 0.35 | 0.22 |
| 5 | 7 | 10 | 0.24 | 0.82 | 0.62 | 0.29 | 0.22 |
| 1 | 5 | 9 | 0.26 | 0.91 | 0.60 | 0.33 | 0.22 |
| 2 | 4 | 5 | 0.22 | 0.86 | 0.63 | 0.29 | 0.22 |
| 2 | 5 | 7 | 0.24 | 0.79 | 0.62 | 0.28 | 0.22 |
| 2 | 9 | 10 | 0.25 | 0.80 | 0.60 | 0.29 | 0.22 |
| 1 | 4 | 5 | 0.23 | 0.93 | 0.62 | 0.32 | 0.22 |
| 3 | 4 | 9 | 0.34 | 0.90 | 0.51 | 0.38 | 0.22 |
| 1 | 9 | 10 | 0.24 | 0.91 | 0.61 | 0.32 | 0.22 |
| 1 | 2 | 9 | 0.26 | 0.84 | 0.57 | 0.31 | 0.21 |
| 5 | 6 | 9 | 0.24 | 0.89 | 0.60 | 0.32 | 0.21 |
| 7 | 8 | 9 | 0.28 | 0.84 | 0.55 | 0.32 | 0.21 |
| 3 | 7 | 9 | 0.32 | 0.89 | 0.52 | 0.36 | 0.21 |
| 2 | 7 | 10 | 0.25 | 0.73 | 0.59 | 0.26 | 0.21 |
| 1 | 4 | 10 | 0.23 | 0.90 | 0.61 | 0.31 | 0.21 |
| 1 | 5 | 7 | 0.22 | 0.89 | 0.62 | 0.30 | 0.21 |
| 2 | 4 | 10 | 0.22 | 0.81 | 0.61 | 0.28 | 0.21 |
| 6 | 9 | 10 | 0.22 | 0.90 | 0.61 | 0.31 | 0.21 |
| 4 | 5 | 6 | 0.21 | 0.93 | 0.63 | 0.31 | 0.21 |
| 2 | 6 | 9 | 0.24 | 0.84 | 0.58 | 0.30 | 0.21 |
| 4 | 7 | 8 | 0.26 | 0.86 | 0.57 | 0.31 | 0.21 |
| 1 | 7 | 10 | 0.23 | 0.84 | 0.59 | 0.29 | 0.21 |
| 1 | 2 | 4 | 0.23 | 0.86 | 0.60 | 0.30 | 0.21 |
| 5 | 6 | 7 | 0.22 | 0.85 | 0.60 | 0.29 | 0.21 |
| 4 | 6 | 10 | 0.21 | 0.90 | 0.62 | 0.30 | 0.21 |
| 3 | 4 | 7 | 0.28 | 0.91 | 0.53 | 0.35 | 0.21 |
| 1 | 6 | 9 | 0.23 | 0.93 | 0.59 | 0.32 | 0.21 |
| 1 | 2 | 7 | 0.23 | 0.81 | 0.59 | 0.28 | 0.21 |
| 6 | 7 | 10 | 0.23 | 0.81 | 0.59 | 0.28 | 0.20 |
| 2 | 4 | 6 | 0.20 | 0.88 | 0.62 | 0.28 | 0.20 |
| 2 | 6 | 7 | 0.22 | 0.79 | 0.59 | 0.27 | 0.20 |
| 1 | 4 | 6 | 0.21 | 0.95 | 0.61 | 0.31 | 0.20 |
| 5 | 8 | 9 | 0.20 | 0.90 | 0.60 | 0.29 | 0.20 |
| 1 | 6 | 7 | 0.21 | 0.89 | 0.59 | 0.29 | 0.20 |
| 2 | 8 | 9 | 0.22 | 0.80 | 0.56 | 0.27 | 0.19 |
| 4 | 5 | 8 | 0.19 | 0.91 | 0.62 | 0.28 | 0.19 |
| 8 | 9 | 10 | 0.22 | 0.83 | 0.57 | 0.28 | 0.19 |
| 3 | 5 | 9 | 0.26 | 0.89 | 0.53 | 0.32 | 0.19 |
| 1 | 8 | 9 | 0.20 | 0.93 | 0.58 | 0.30 | 0.19 |
| 4 | 8 | 10 | 0.22 | 0.80 | 0.56 | 0.27 | 0.19 |
| 2 | 5 | 10 | 0.17 | 0.73 | 0.62 | 0.22 | 0.19 |
| 3 | 4 | 5 | 0.23 | 0.90 | 0.54 | 0.31 | 0.19 |
| 3 | 9 | 10 | 0.24 | 0.89 | 0.53 | 0.31 | 0.19 |
| 2 | 3 | 9 | 0.26 | 0.82 | 0.51 | 0.30 | 0.19 |
| 2 | 4 | 8 | 0.20 | 0.82 | 0.58 | 0.26 | 0.19 |
| 1 | 5 | 10 | 0.15 | 0.91 | 0.67 | 0.25 | 0.19 |
| 1 | 4 | 8 | 0.20 | 0.92 | 0.58 | 0.29 | 0.19 |
| 5 | 7 | 8 | 0.21 | 0.78 | 0.56 | 0.26 | 0.19 |
| 1 | 2 | 5 | 0.18 | 0.80 | 0.61 | 0.24 | 0.19 |
| 1 | 3 | 9 | 0.24 | 0.93 | 0.52 | 0.33 | 0.19 |
| 6 | 8 | 9 | 0.20 | 0.90 | 0.58 | 0.29 | 0.19 |
| 5 | 6 | 10 | 0.16 | 0.85 | 0.64 | 0.24 | 0.18 |
| 3 | 5 | 7 | 0.21 | 0.89 | 0.55 | 0.29 | 0.18 |
| 7 | 8 | 10 | 0.26 | 0.67 | 0.50 | 0.25 | 0.18 |
| 2 | 5 | 6 | 0.17 | 0.78 | 0.61 | 0.23 | 0.18 |
| 2 | 7 | 8 | 0.23 | 0.69 | 0.53 | 0.24 | 0.18 |
| 3 | 4 | 10 | 0.23 | 0.87 | 0.53 | 0.30 | 0.18 |
| 3 | 6 | 9 | 0.23 | 0.92 | 0.52 | 0.32 | 0.18 |
| 4 | 6 | 8 | 0.18 | 0.91 | 0.59 | 0.28 | 0.18 |
| 2 | 3 | 4 | 0.22 | 0.84 | 0.52 | 0.29 | 0.18 |
| 1 | 5 | 6 | 0.15 | 0.93 | 0.63 | 0.26 | 0.18 |
| 1 | 3 | 4 | 0.23 | 0.92 | 0.52 | 0.32 | 0.18 |
| 3 | 7 | 10 | 0.22 | 0.83 | 0.52 | 0.28 | 0.18 |
| 2 | 3 | 7 | 0.22 | 0.80 | 0.53 | 0.27 | 0.18 |
| 1 | 7 | 8 | 0.21 | 0.81 | 0.54 | 0.27 | 0.18 |
| 1 | 2 | 10 | 0.17 | 0.75 | 0.59 | 0.22 | 0.18 |
| 2 | 6 | 10 | 0.17 | 0.73 | 0.59 | 0.21 | 0.18 |
| 3 | 4 | 6 | 0.20 | 0.93 | 0.53 | 0.30 | 0.17 |
| 1 | 6 | 10 | 0.15 | 0.90 | 0.63 | 0.25 | 0.17 |
| 6 | 7 | 8 | 0.21 | 0.77 | 0.52 | 0.25 | 0.17 |
| 1 | 3 | 7 | 0.20 | 0.92 | 0.53 | 0.30 | 0.17 |
| 1 | 2 | 6 | 0.16 | 0.81 | 0.59 | 0.24 | 0.17 |
| 3 | 6 | 7 | 0.20 | 0.89 | 0.53 | 0.29 | 0.17 |
| 2 | 5 | 8 | 0.16 | 0.69 | 0.56 | 0.20 | 0.16 |
| 5 | 8 | 10 | 0.16 | 0.72 | 0.56 | 0.21 | 0.16 |
| 1 | 5 | 8 | 0.13 | 0.90 | 0.62 | 0.23 | 0.16 |
| 3 | 8 | 9 | 0.20 | 0.90 | 0.50 | 0.29 | 0.16 |
| 5 | 6 | 8 | 0.14 | 0.81 | 0.57 | 0.22 | 0.16 |
| 2 | 3 | 5 | 0.17 | 0.77 | 0.52 | 0.23 | 0.16 |
| 3 | 5 | 10 | 0.15 | 0.86 | 0.56 | 0.24 | 0.16 |
| 1 | 2 | 8 | 0.16 | 0.72 | 0.53 | 0.21 | 0.15 |
| 2 | 8 | 10 | 0.20 | 0.56 | 0.48 | 0.18 | 0.15 |
| 3 | 4 | 8 | 0.19 | 0.87 | 0.49 | 0.27 | 0.15 |
| 1 | 8 | 10 | 0.16 | 0.75 | 0.53 | 0.22 | 0.15 |
| 1 | 3 | 5 | 0.15 | 0.94 | 0.55 | 0.26 | 0.15 |
| 2 | 6 | 8 | 0.16 | 0.68 | 0.52 | 0.20 | 0.15 |
| 3 | 5 | 6 | 0.15 | 0.89 | 0.54 | 0.25 | 0.15 |
| 6 | 8 | 10 | 0.17 | 0.69 | 0.51 | 0.20 | 0.15 |
| 1 | 6 | 8 | 0.13 | 0.87 | 0.56 | 0.23 | 0.15 |
| 2 | 3 | 10 | 0.17 | 0.70 | 0.49 | 0.21 | 0.15 |
| 1 | 2 | 3 | 0.17 | 0.80 | 0.50 | 0.23 | 0.15 |
| 1 | 3 | 10 | 0.14 | 0.90 | 0.54 | 0.24 | 0.15 |
| 3 | 7 | 8 | 0.19 | 0.80 | 0.46 | 0.25 | 0.15 |
| 2 | 3 | 6 | 0.16 | 0.78 | 0.50 | 0.23 | 0.15 |
| 3 | 6 | 10 | 0.14 | 0.86 | 0.53 | 0.23 | 0.14 |
| 1 | 3 | 6 | 0.14 | 0.95 | 0.53 | 0.25 | 0.14 |
| 3 | 5 | 8 | 0.13 | 0.83 | 0.49 | 0.21 | 0.13 |
| 2 | 3 | 8 | 0.16 | 0.67 | 0.42 | 0.19 | 0.12 |
| 1 | 3 | 8 | 0.12 | 0.88 | 0.47 | 0.22 | 0.12 |
| 3 | 8 | 10 | 0.16 | 0.68 | 0.41 | 0.20 | 0.12 |
| 3 | 6 | 8 | 0.13 | 0.81 | 0.45 | 0.21 | 0.12 |
